# Unilateral Ulnar Nerve Instability After Bilateral Surgical Transposition of the Medial Triceps and Ulnar Nerve

**DOI:** 10.1155/cro/4793039

**Published:** 2026-09-15

**Authors:** Thilo Schmitt, Jean-Michel Hovsepian, Stephan Vogt

**Affiliations:** ^1^ Department of Orthopedics and Orthopedic Surgery, Hessing Stiftung, Augsburg, Germany; ^2^ Department of Orthopedics and Orthopedic Surgery, University of Augsburg, Augsburg, Germany, uni-augsburg.de

## Abstract

Medial snapping elbow is a rare condition. It may be caused by a hypermobile portion of the medial triceps, a hypermobile ulnar nerve, or a combination of both. In this report, we present a 21‐year‐old male patient with bilateral combined snapping elbow. The hypermobile portion of the triceps was identified and transposed in combination with anterior transposition of the ulnar nerve on both sides. At follow‐up, the patient demonstrated recurrent snapping at 70° of flexion corresponding to recurrent instability of the left ulnar nerve, although identical surgical procedures had been performed bilaterally. Revision surgery with a fascial sling was discussed for the left elbow; however, the patient was satisfied with the outcome and declined further intervention.

## 1. Introduction

Medial snapping elbow is a rare condition [1]. It typically occurs without structural damage and may be caused by a hypermobile portion of the medial triceps, a hypermobile ulnar nerve, or both. With increasing elbow flexion, the ulnar nerve displaces anteriorly from the sulcus ulnaris at approximately 70° of flexion; the medial portion of the triceps follows at approximately 100° [2]. Radiographs and nerve conduction studies are usually normal. MRI may demonstrate nerve edema. The diagnosis is primarily clinical and can be confirmed by dynamic ultrasound. Conservative treatment may reduce symptoms such as pain [3]; however, definitive management is surgical. Several techniques have been described, including ulnar nerve transposition and transposition or resection of the medial portion of the triceps tendon [4].

## 2. Case Report

**Figure 1:**
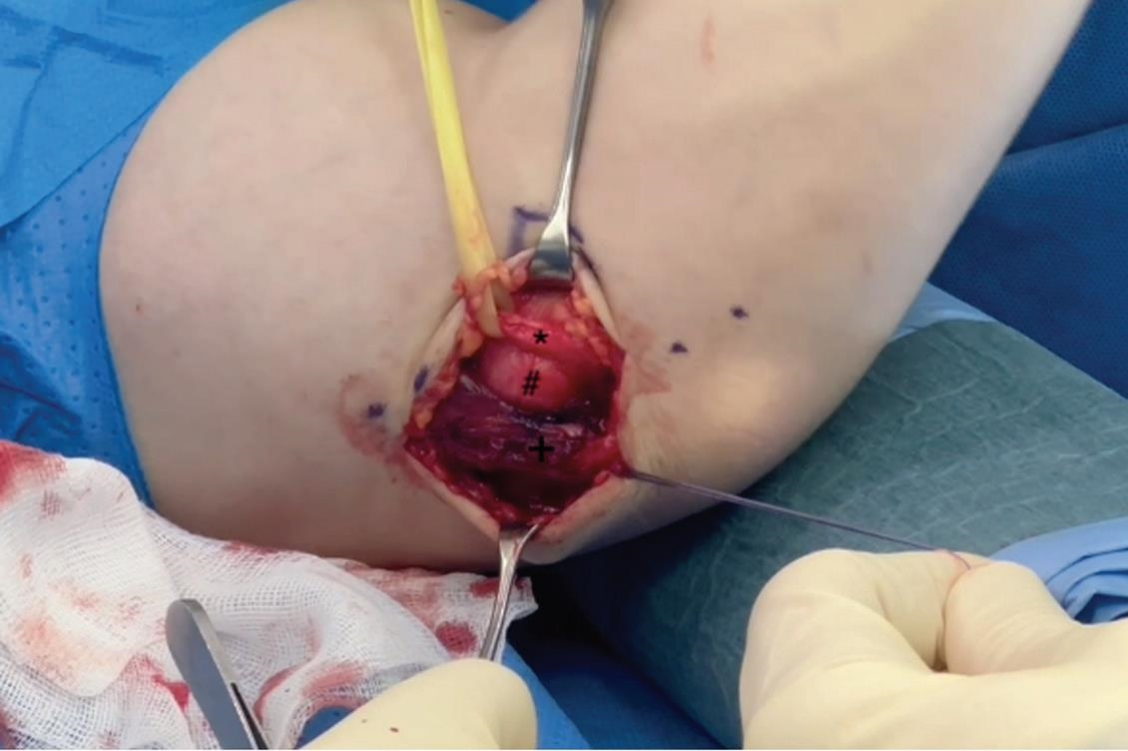
Intraoperative picture of the left elbow. “∗”: mobilized ulnar nerve anterior to the ulnar epicondyle. “#”: ulnar epicondyle. “+”: test‐reposition of the snapping part of the triceps, which is shuttled laterally through the muscle.

**Figure 2:**
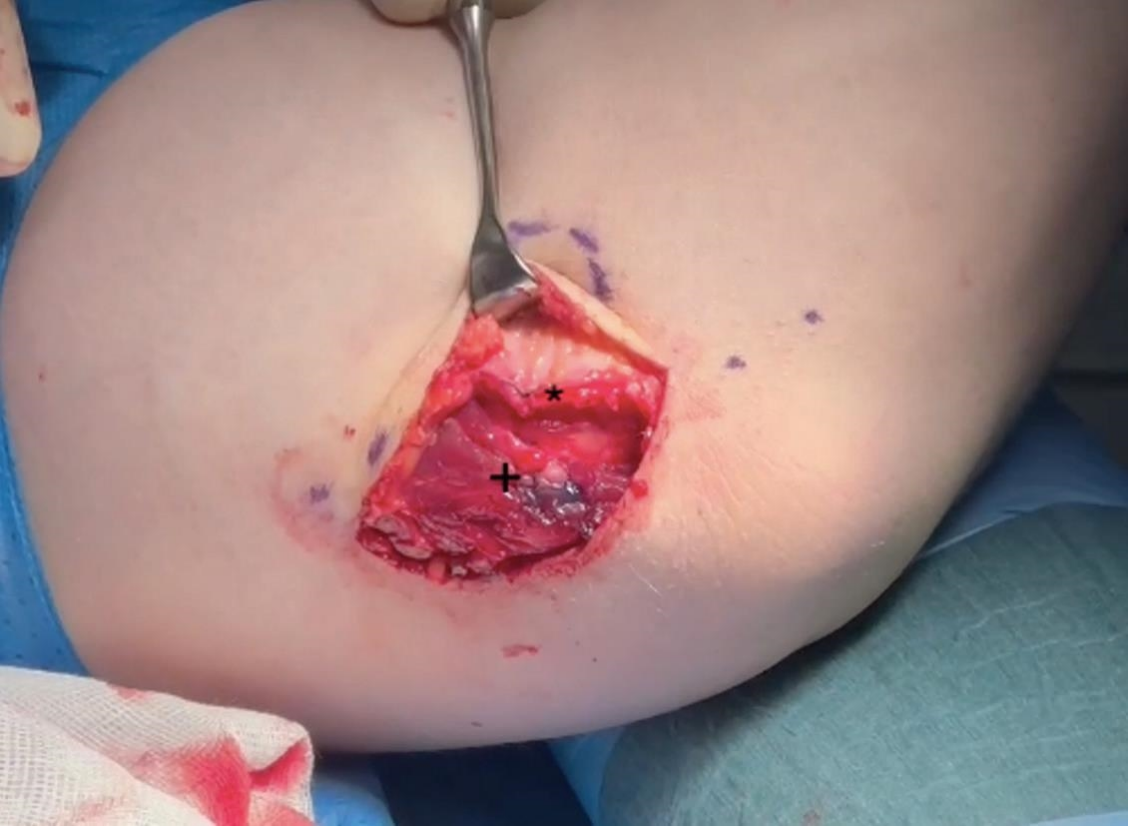
Intraoperative picture of the left elbow in flexion. “∗”: adipose flap with the underlying ulnar nerve. “+”: fixated triceps flap.

A healthy 21‐year‐old male patient presented to our hospital with repeated loud snapping in both elbows during strength training. The symptoms interfered with his daily fitness routine required for his occupation as a police officer, and he sought therapeutic options. Clinical examination revealed stable, pain‐free elbows with full active and passive range of motion. In the supine position, with the shoulder abducted to 90° and externally rotated, both elbows demonstrated snapping at 70° and 100° of flexion [2], which increased with resistance. Radiographs and MRI of both elbows were unremarkable, and no edema of the ulnar nerve was detected. Nerve conduction studies were normal bilaterally. After discussion with the patient, surgical treatment was planned, beginning with the dominant right side. The procedure was performed under general anesthesia with the patient supine and the arm abducted and externally rotated. A dorsal incision was made over the ulnar epicondyle. After subcutaneous dissection, intraoperative dynamic assessment with direct visualization confirmed subluxation of the ulnar nerve at 70° of flexion and subluxation of the medial triceps at 100°. Myoelectric stimulation was not required. The ulnar nerve was neurolysed and secured with a vascular loop (Figure 1). During dynamic assessment, the hypermobile portion of the triceps was identified and marked. It was mobilized from the ulna to create a proximally based flap of the medial triceps. A central intramuscular incision was made, and the flap was passed from deep to superficial through the triceps and fixed with nonabsorbable sutures. The ulnar nerve was then transposed anteriorly and secured with a subcutaneous adipose flap (Figure 2) [5].

Given the patient′s occupational demands, we aim to preserve functional structures as much as possible. Therefore, we preferred triceps flap transposition over resection and avoided creating a fascial sling from the intact flexor fascia.

Postoperative rehabilitation included immobilization in a cast at 90° of flexion for 2 weeks. Active motion was initiated 6 weeks postoperatively. At 3 months, the patient was pain‐free, had full range of motion, and reported no snapping. Surgery on the contralateral side was performed 9 months later using the same technique and rehabilitation protocol. In contrast to the right side, the patient returned 3 months postoperatively due to recurrent snapping on the left side. Clinical examination revealed snapping at 70° of flexion, corresponding to the intraoperative finding of anterior ulnar nerve subluxation. Snapping of the medial triceps at 100° was neither observed nor reported. Further imaging (dynamic ultrasound or MRI) and revision surgery with ulnar nerve exploration and stabilization using a fascial sling of the flexor fascia were discussed [6]. However, the patient declined further intervention, as the residual snapping did not interfere with his daily or professional activities. A follow‐up assessment was conducted by interviewing the patient 30 months postoperatively on the right side and 22 months postoperatively on the left side. In addition, a MEPS score was recorded for each side. The maximum achievable score with 100 out of 100 was reported for the right side. Regarding the left elbow the score was 85 out of 100, the maximum score was achieved in the categories of mobility, stability, and function. Regarding pain, the patient reported a score of 30 out of 45. The patient also stated that the symptoms were occasional and mild (Table 1).

**Table 1:** MEPS score for left (FU 22 m) and right elbow (FU 30 m).

Function	Points	Definition (points)	Elbow L	Elbow R
Pain	45	None (45)	30	45
		Mild (30)		
		Moderate (15)		
		Severe (0)		
Motion	20	Arc > 100° (20)	20	20
		Arc 50°–100° (15)		
		Arc < 50° (5)		
Stability	10	Stable (10)	10	10
		Moderate instability (5)		
		Gross instability (0)		
Function	25	Comb hair (5)	25	25
		Feed (5)		
		Perform hygiene (5)		
		Don shirt (5)		
		Don short (5)		
Total	100		85	100

*Note:* Classification: excellent, > 90; good, 75–89; fair, 60–74; poor, < 60.

## 3. Discussion

This case describes bilateral medial snapping elbow treated with identical surgical procedures: transposition of the snapping medial triceps portion and anterior transposition of the ulnar nerve secured with a subcutaneous flap. On the nondominant left side, recurrent ulnar nerve instability occurred and was managed conservatively without functional limitation. Stabilization of the ulnar nerve using a fascial sling of the flexor muscles might have provided greater stability and prevented recurrence. We believe that patients are primarily symptomatic dominantly from triceps snapping, which increases with triceps contraction, whereas ulnar nerve snapping does not increase with muscle contraction and may therefore be better tolerated.

## 4. Conclusion

This case report demonstrates successful bilateral surgical treatment of snapping triceps by transposition, with recurrent ulnar nerve instability on the nondominant side after stabilization with an adipose flap. The adipose flap was chosen to minimize disruption of the intact medial flexor fascia in this highly active patient. However, the compromise in stability led to recurrent ulnar nerve instability. The residual instability was accepted without revision surgery and did not impair daily or professional elbow function. The patient remained satisfied with the outcome and returned to full field service as a police officer. Based on this experience, we recommend using a fascial sling in similar cases to ensure secure stabilization of the ulnar nerve and to optimize outcomes.

## Funding

No funding was received for this manuscript.

## Ethics Statement

This case report was approved by the chairman of the Ethics Committee of the Hessing Stiftung Augsburg (1/25). Informed consent was obtained from the patient for the publication of this report and any accompanying images. The patient′s anonymity has been preserved throughout the manuscript.

## Conflicts of Interest

The authors declare no conflicts of interest.

## Data Availability

The data that support the findings of this study are available on request from the corresponding author. The data are not publicly available due to privacy or ethical restrictions.
